# Author Correction: Epitope-directed monoclonal antibody production using a mixed antigen cocktail facilitates antibody characterization and validation

**DOI:** 10.1038/s42003-021-02091-4

**Published:** 2021-05-04

**Authors:** Oi Wah Liew, Samantha S. M. Ling, Shera Lilyanna, Yue Zhou, Peipei Wang, Jenny P. C. Chong, Yan Xia Ng, Angeline E. S. Lim, Eliot R. Y. Leong, Qifeng Lin, Teck Kwang Lim, Qingsong Lin, Enoch M. W. Ng, Tuck Wah Ng, A. Mark Richards

**Affiliations:** 1 Cardiovascular Research Institute, Department of Medicine, Yong Loo Lin School of Medicine, National University of Singapore, National University Health System, Singapore, Singapore; 2 Department of Biological Sciences, National University of Singapore, Singapore, Singapore; 3 Laboratory for Optics & Applied Mechanics, Department of Mechanical & Aerospace Engineering, Monash University, Clayton, VIC Australia; 4 Christchurch Heart Institute, University of Otago, Christchurch, New Zealand

**Keywords:** ELISA, Immunoblotting, Immunohistochemistry, Immunoprecipitation, Antibody generation

Correction to: *Communications Biology* 10.1038/s42003-021-01965-x, published online 06 April 2021.

The original version of this Article contained an error in the spelling of the author Qingsong Lin, which was incorrectly given as Qinsong Lin. This has now been corrected in both the PDF and HTML versions of the Article.

