# Supplementary material for: Epitope-directed monoclonal antibody production using a mixed antigen cocktail facilitates antibody characterization and validation
Source: Commun Biol. 2021 Apr 6;4:441. doi: 10.1038/s42003-021-01965-x (PMC8024308; doi:10.1038/s42003-021-01965-x)
Supplement: Supplementary file 2 — Supplementary Information [file 42003_2021_1965_MOESM2_ESM.pdf]

SUPPLEMENTARY INFORMATION FOR:

Epitope-Directed Monoclonal Antibody Production Using A Mixed Antigen Cocktail Facilitates Antibody Characterization And Validation

Oi Wah Liew[#1], Samantha S. M. Ling[#1], Shera Lilyanna[1], Yue Zhou[1], Peipei Wang[1], Jenny P.C. Chong[1], Yan Xia Ng[1], Angeline E.S Lim[1], Eliot R.Y. Leong[1], Qifeng Lin[4], Teck Kwang Lim[4], Qinsong Lin[4], Enoch M.W. Ng[3], Tuck Wah Ng[3], A. Mark Richards[1,2]*

[1] Cardiovascular Research Institute, Dept of Medicine, Yong Loo Lin School of Medicine, National University of Singapore, National University Health System, Singapore, Singapore.

[2] Christchurch Heart Institute, University of Otago, Christchurch, New Zealand

[3] Laboratory for Optics & Applied Mechanics, Department of Mechanical & Aerospace Engineering, Monash University, Clayton, VIC, 3800, Australia.

[4] Department of Biological Sciences, National University of Singapore, Singapore, Singapore

Supplementary Note 1. Recombinant Expression and Purification of Thioredoxin-fused AG1, AG4 and AG5

The thioredoxin tripeptide gene constructs were expressed in *E. coli* strain BL21 (DE3) *trx*B and bacteria pellets were processed to obtain total, soluble and insoluble protein fractions. Images of the expression profile after separation by SDS-PAGE was acquired with the ChemiDoc™ Touch Imaging System (BioRad, Hercules, CA, USA). Band sizing and intensity analyses with global lane background subtraction are determined using the MW and Quantity tools in the Image Lab software. All expressed proteins were found to migrate at higher molecular weight positions on SDS-PAGE than their respective theoretical masses. This anomaly is typical of the electrophoretic behavior of thioredoxin fusion proteins as in previous published reports[1-3].

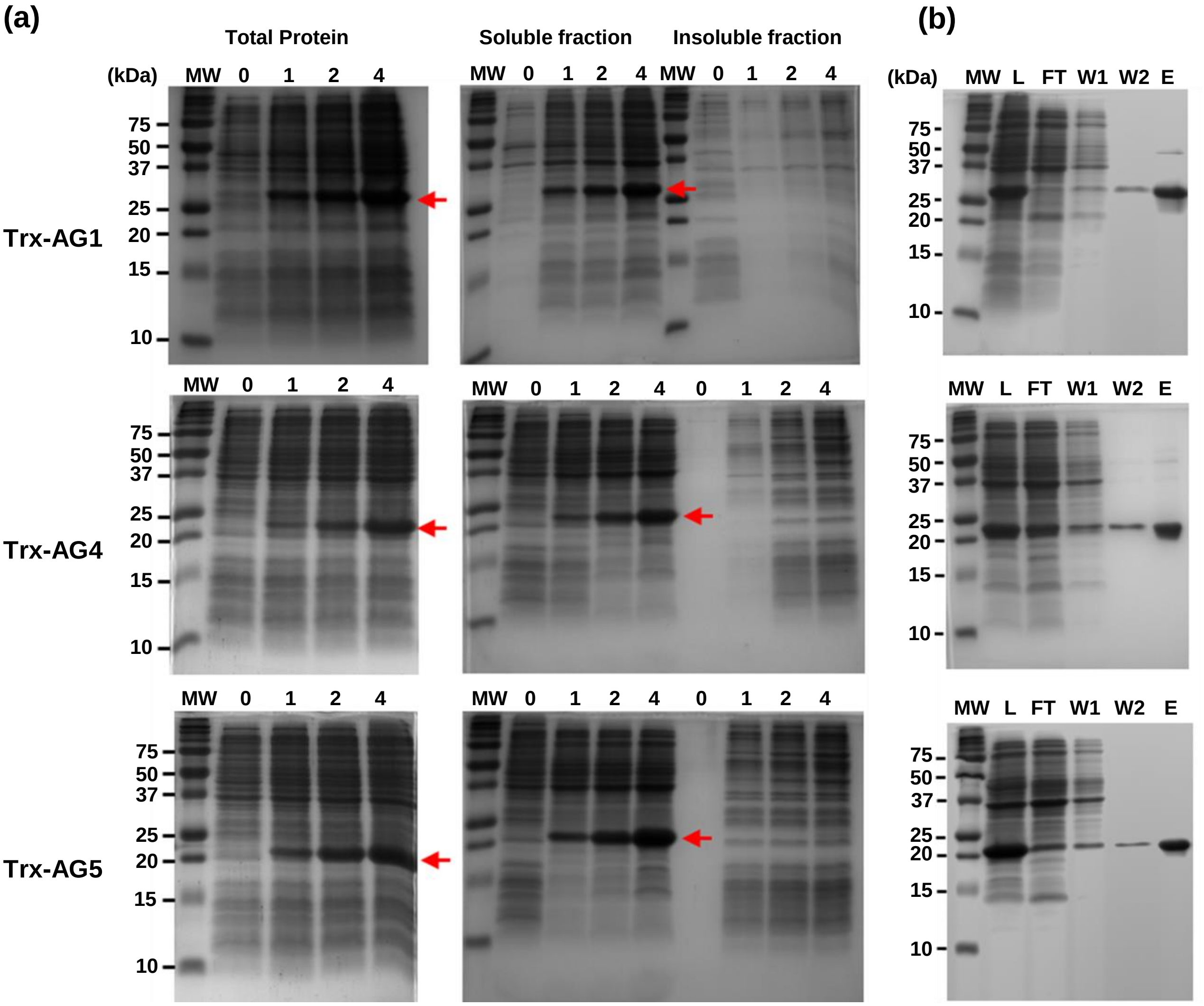

Supplementary Figure 1. Antigen production in *E. coli*. (a) SDS-PAGE (16%) profile of total, soluble and insoluble cellular proteins isolated from *E. coli* strain BL21 (DE3) *trxB* expressing Trx-AG1, -AG4 and -AG5. Samples were collected at 0, 1, 2 and 4 h after IPTG induction. The induced proteins are indicated with an arrow. **(b)** Purification of Trx-AG1, -AG4 and –AG5 using the "Native + Desalting" method on the Profinia Protein Purification system. SDS-PAGE profile of IMAC fractions: (L) supernatant cell lysate; (F) column flow-through; (W1) Wash 1; (W2) Wash 2; (ED) Desalted elution. Abbreviation: MW, Protein molecular weight ladder (Precision Plus Protein™ All Blue Standards, Bio-Rad).

Supplementary Table 1. Physicochemical properties of antigens. Predictions are from the Protparam tool on the ExPASy Molecular Biology Server (www.expasy.org) and bacterial expression characteristics of thioredoxin-fused antigens. The instability index may be taken as a measure of the *in vivo* half-life of a protein, where values >40 predicts an unstable protein. The aliphatic index serves as a measure of the thermostability of a protein where high values are indicative of stability over a wide temperature range. The GRAVY value indicates the propensity of the protein to interact with water, the lower the value the greater the hydrophilicity of the protein.

Parameter \ Protein	**Trx-AG1**	**Trx-AG4**	**Trx-AG5**
No. of residues	207	174	177
Theoretical Molecular Mass (Da)	21946	18501	18709
Theoretical pI	4.70	4.64	5.21
Instability Index	2.39	7.44	3.28
Aliphatic Index	61.35	76.26	78.36
Grand Average Hydropathicity (GRAVY)	-0.691	-0.476	-0.541
Molecular Mass by SDS-PAGE (kDa)	27.2	22.1	20.5
Expression level (% of total bacterial proteins)	20.3	26.2	29.6
% Soluble protein (relative to total expressed protein)	99.5	99.4	96.9
Yield of purified protein (mg per L shake flask culture)	40.8	39.7	37.7

Supplementary Note 2. DEXT Microplate Evaporative Losses

Evaporation of droplets is easily controlled by placement of the DEXT microplates on a stand within an air-tight box enclosure with a layer of water at the bottom to generate a full moisture-saturated environment. A bespoke microplate lid was designed with a microfiber cloth affixed on one side. When the microfiber cloth is pre-wetted with water and the lid then inverted over the samples, this further reduces evaporation loss of samples from the wells. The efficacy of the evaporation reduction elements was tested by dispensing 15 μL of deionized water onto the wells of three sheets. One sheet was housed inside the microplate base without a lid, with a lid with dry microfiber cloth, or a lid with a microfibre cloth pre-wetted with water inverted over it. All were placed sequentially in a refrigerated environment at 4°C for 8 hours, followed by placement in the ambient environment (24°C, 55%RH) for 3 h, and finally into an incubator set at 37°C for 1 hour. Measurement was done by using a capillary tube of 0.68 mm inner diameter to drain the liquid in each well into it. The volume was determined via measurement of the length of liquid in the tube and its known inner cross sectional area. The liquid in the sheet wells housed in the base without a lid were mostly evaporated, and these were thus not measured.

Supplementary Figure 2a presents the results of the test which indicated that the residual liquid volumes had mean and standard deviation values of 8.68 μL and 0.51 μL respectively with the lid alone. Using the pre-wetted lid these values were alternatively 14.16 μL and 0.41 μL respectively. It should be noted that in transferring liquid from the well to the capillary tube for volume measurement, some liquid will inevitably be retained in the well due to the wetting characteristics of real surfaces. This implies that the actual evaporative losses from the wells may in fact be lower. Notwithstanding, using the pre-wetted lid resulted in approximately 7.5 times less likelihood of evaporation losses over just using the lid alone. In addition, the standard deviation over mean ratios (0.06 for lid and 0.03 for lid with pre-wetted microfiber cloth) imputed lower relative spread of volumes when normalized. Taken together, the use of a pre-wetted lid is highly effective in minimizing evaporative losses even over long incubation times and under challenging environmental conditions. Thus, the adverse effects of liquid evaporative losses on assay uniformity will be minimal. On inspecting the distribution of residual liquid volumes across the DEXT microplate (**Supplementary Figure 2b and c**), no discernible edge effect was observed regardless of whether the lid had a pre-wetted microfibre cloth or not. This

may be attributed to the direct heating mode of the wells on the sheet and the flat architecture which reduces heat loss through the sides[4].

(a)

.

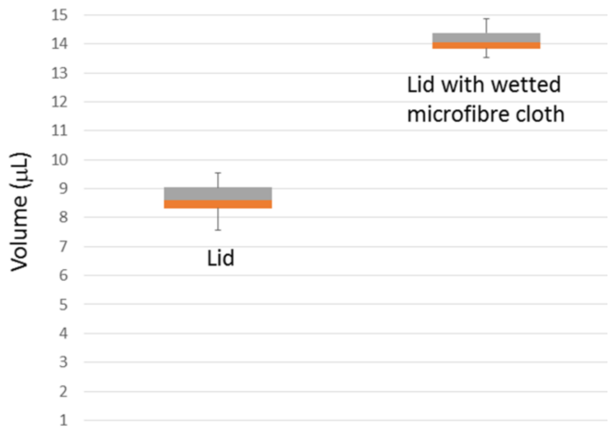

(b)

	A	B	C	D	E	F	G	H	I	J	K	L
1	8.09		8.53		8.46		8.20		8.28		8.05	
2												7.57
3	9.40		8.88		8.90		7.93		8.34			
4										8.15		9.26
5	9.49		9.54		9.45		8.86					
6				8.56		9.09		9.42		8.41		8.75
7	8.68											
8		8.70		8.57		8.54		8.32		8.63		9.23

(c)

	A	B	C	D	E	F	G	H	I	J	K	L
1	13.82		13.79		14.50		14.29		13.68		14.30	
2												13.53
3	13.89		14.87		14.74		14.03		14.87			
4										14.86		14.03
5	14.06		14.36		13.80		13.93					
6				13.92		14.07		14.71		14.38		14.38
7	14.16											
8		14.66		14.17		13.81		13.53		13.54		14.00

Supplementary Figure 2. Performance of DEXT microplates against evaporation. (a) Box plots of volume measurements of residual liquid in DEXT microplate wells (n=30; original liquid volume = 15µl per well) after storage in a refrigerated environment at 4°C for 8 hours, followed by placement in the ambient environment (24°C, 55%RH) for 3 hours and finally into an incubator set at 37°C for 1 hour. Sheets are placed inside the bespoke microplate base and a lid with and without a pre-wetted microfibre cloth is inverted over the sheets. The ability for evaporation resistance is clearly demonstrated in the former. The distribution of volume measurements across the DEXT microplate sheet covered by a lid without **(b)** or with **(c)** a pre-wet microfiber cloth is shown.

Supplementary Note 3. Determination of mAb binding affinity

SPR analysis showed two mAbs (AG4/13E10 and 4E1) that interacted with its cognate thioredoxin-fused antigen but did not bind to recombinant hANKRD1 (OriGene Technologies). Both mAbs were directed towards the AG4 sequence which is located internally with respect to the primary hANKRD1 sequence. Anti-AG1 and -AG5 mAbs appear to be susceptible to acid treatment for regenerating the sensor chips as depicted by loss of dose-response against their cognate thioredoxin-fused antigen.

Supplementary Figure 3. Antibody binding to full-length hANKRD1 and its cognate peptide fragments by SPR analysis. SPR binding interaction of immobilized mAbs raised against **(a)** AG1, **(b)** AG4 and **(c)** AG5 are shown. The antibodies were first tested against recombinant hANKRD1 and were subsequently interacted with their cognate thioredoxin-fused antigen after sensor surface regeneration. Each plot depicts purified antibody from one hybridoma clone interacting with 5 different concentrations of analyte at 270, 90, 30, 10 and 3.33 nM. The phosphate buffer reference blank is designated the 0nM lane.

(a) AG1 mAbs

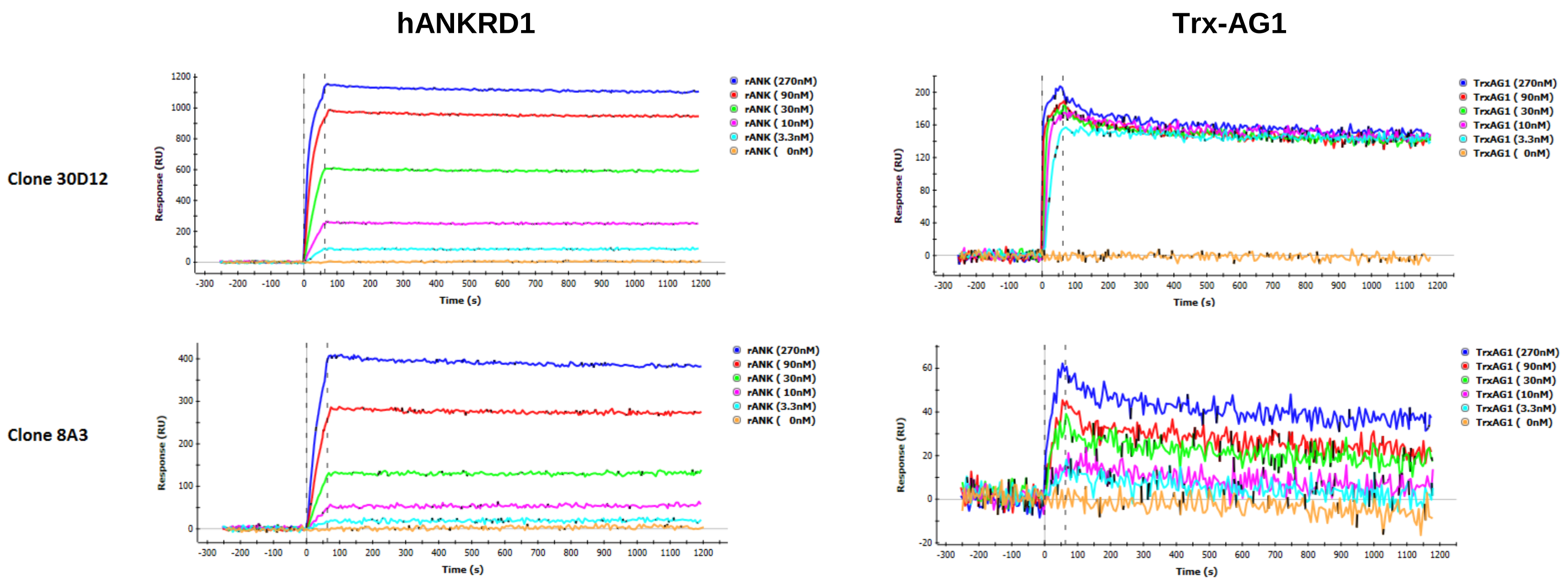

(b) AG4 mAbs

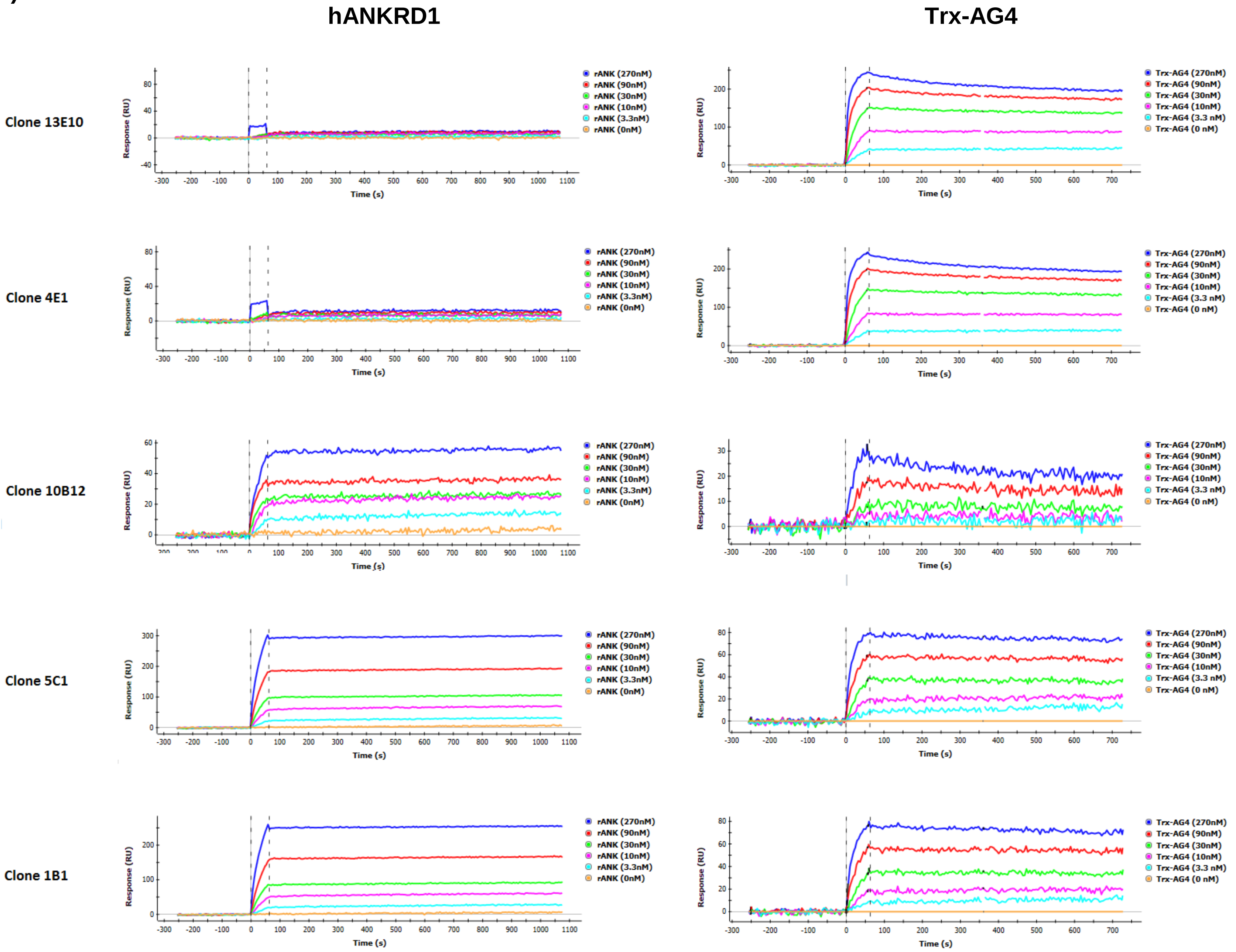
hANKRD1
Trx-AG4
Clone 13E10
Clone 4E1
Clone 10B12
Clone 5C1
Clone 1B1
Response (RU)
Time (s)
rANK (270nM)
rANK (90nM)
rANK (30nM)
rANK (10nM)
rANK (3.3nM)
rANK (0nM)
Trx-AG4 (270nM)
Trx-AG4 (90nM)
Trx-AG4 (30nM)
Trx-AG4 (10nM)
Trx-AG4 (3.3 nM)
Trx-AG4 (0 nM)

(c) AG5 mAbs

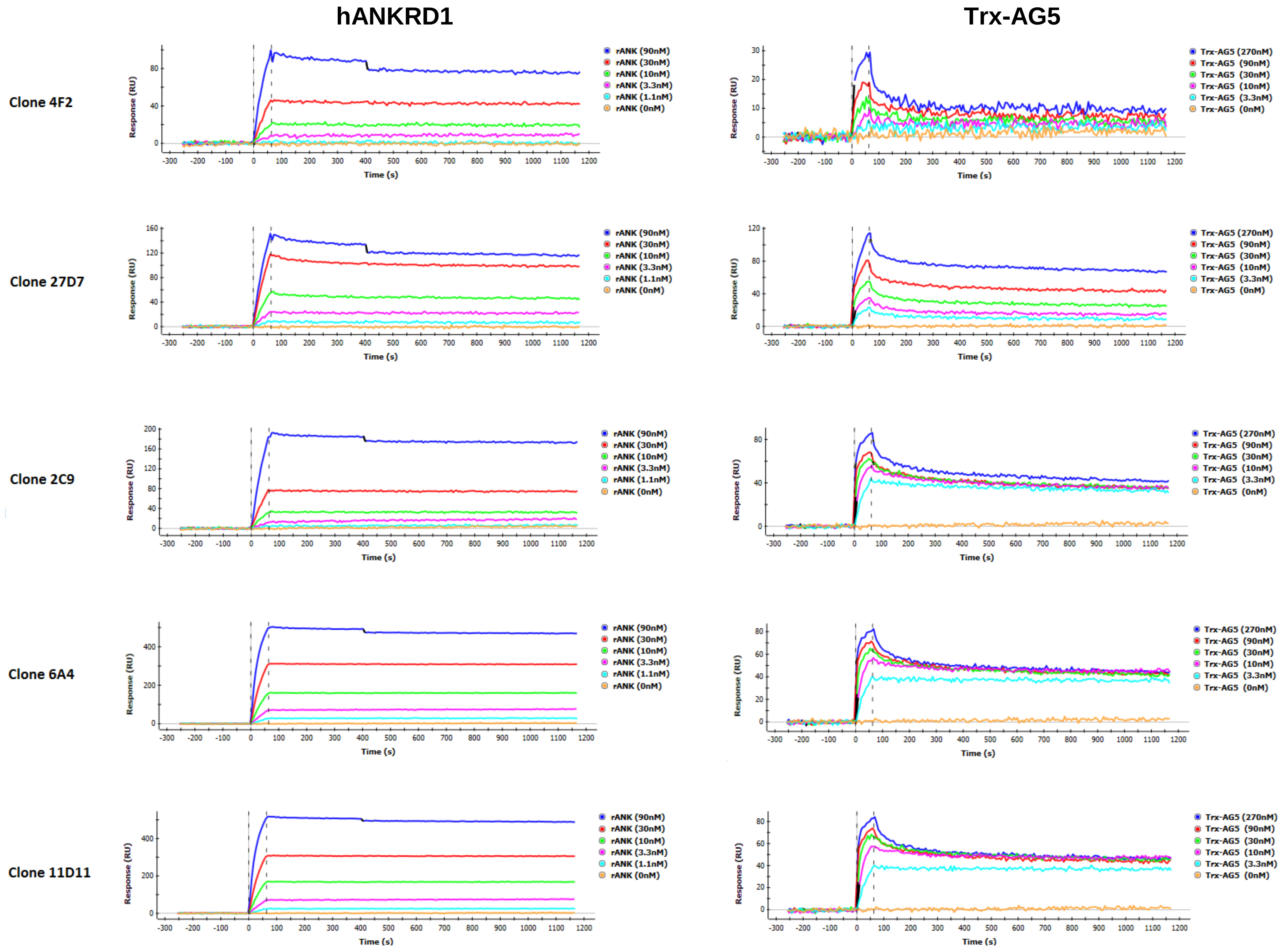
hANKRD1
Trx-AG5
Clone 4F2
Clone 27D7
Clone 2C9
Clone 6A4
Clone 11D11
rANK (90nM)
rANK (30nM)
rANK (10nM)
rANK (3.3nM)
rANK (1.1nM)
rANK (0nM)
Trx-AG5 (270nM)
Trx-AG5 (90nM)
Trx-AG5 (30nM)
Trx-AG5 (10nM)
Trx-AG5 (3.3nM)
Trx-AG5 (0nM)
Response (RU)
Time (s)

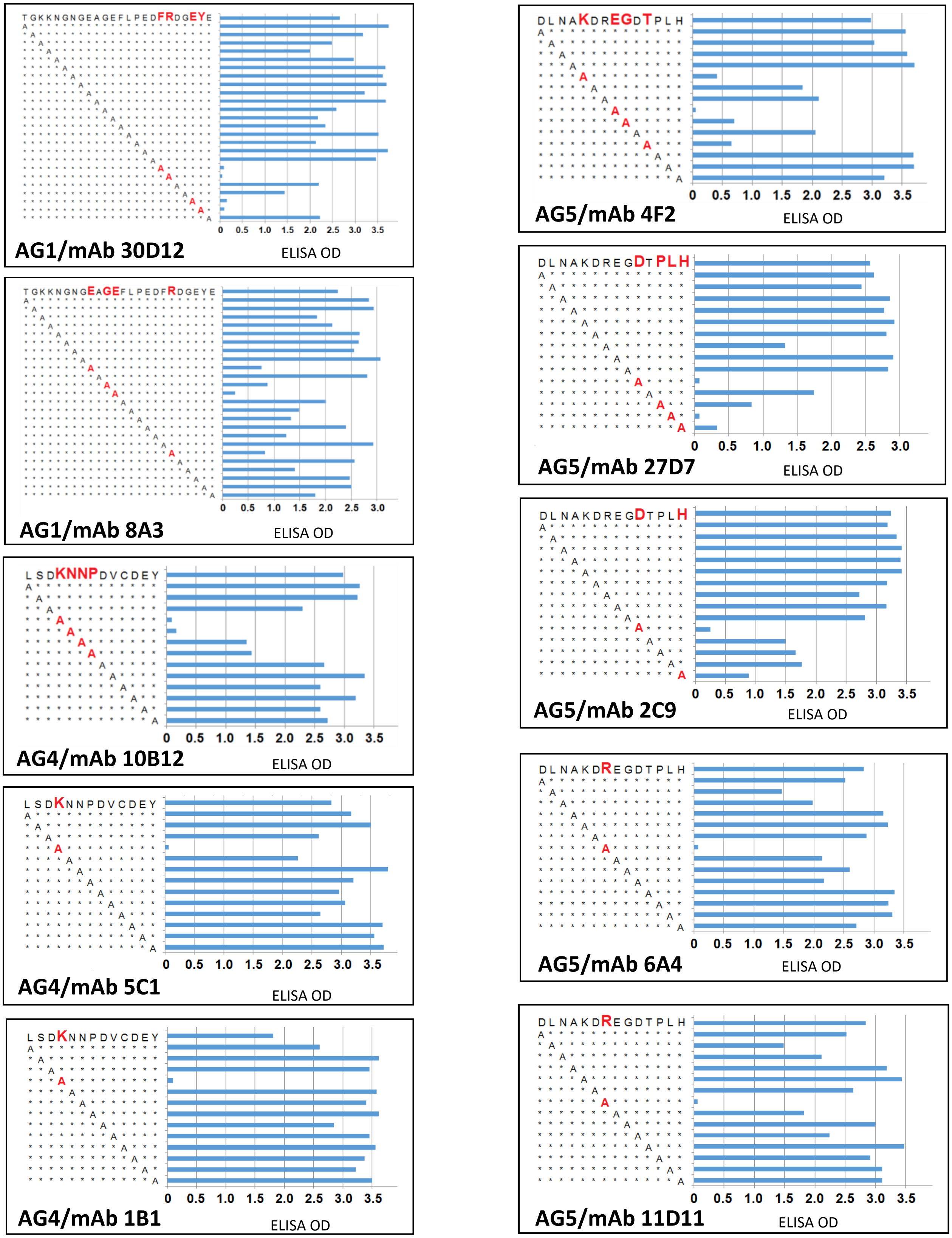

Supplementary Figure 4. Epitope mapping by alanine-scan mutagenesis of mAb binding site. Critical residues (highlighted in red) are defined as those where alanine substitution results in >50% loss of ELISA optical density (OD) reading compared with the wild-type peptide.

```
                10         20         30         40         50         60
Human    MMVLKVEELV TGKKNGNGEA GEFLPEDFRD GEYEAAVTLE KQEDLKTLLA HPVTLGEQQW
Rat      MMVFRVEELV TGKKNSNGSS GEFLPGEFRN GEYEAAVALE KQEDLKTLPA NSVNLGEEQR
         ***::***** *****.**   ***** :**: *******:** ******** * : *.***:*
                                 AG1

                70         80         90        100        110        120
Human    KSEKQREAEL KKKKLEQRSK LENLEDLEII IQLKKRKKYR KTKVPVVKEP EPEIITEPVD
Rat      KSEKVREAEL KKKKLEQRSK LENLEDLEII VQLKKRKKYK KTKVPVVKEP EPEIITEPVD
         **** ***** ********** ********** :********: ********** **********

               130        140        150        160        170        180
Human    VPTFLKAALE NKLPVVEKFL SDKNNPDVCD EYKRTALHRA CLEGHLAIVE KLMEAGAQIE
Rat      VPRFLKAALE NKLPVVEKFL SDKNSPDVCD EYKRTALHRA CLEGHLAIVE KLMEAGAQIE
         ** ******* ********** **** ***** ********** ********** **********
                                  AG4

               190        200        210        220        230        240
Human    FRDMLESTAI HWASRGGNLD VLKLLLNKGA KISARDKLLS TALHVAVRTG HYECAEHLIA
Rat      FRDMLESTAI HWACRGGNLD VLKLLLNKGA KISARDKLLS TALHVAVRTG HYECAEHLIA
         ********** ***.****** ********** ********** ********** **********

               250        260        270        280        290        300
Human    CEADLNAKDR EGDTPLHDAV RLNRYKMIRL LIMYGADLNI KNCAGKTPMD LVLHWQNGTK
Rat      CEADLNAKDR EGDTPLHDAV RLNRYKMIRL LMTFGADLNV KNCAGKTPMD LVLHWQNGTK
         ********** ********** ********** *: :****** ********** **********
                    AG5

               310      319
Human    AIFDSLRENS YKTSRIATF
Rat      AIFDSLKENA YKNSRIATF
         ******:**: **.******
```

Supplementary Figure 5. Cross-species reactivity of ANKRD1 mAbs and validation of mAb-ANKRD1 interaction by immunoprecipitation mass spectrometry (IP-MS). The human and rat ANKRD1 amino acid sequences are aligned using Clustal Omega[5] available from the EMBL-EBI server[6] at https://www.ebi.ac.uk. An asterisk indicates fully conserved residues, blank indicates non-conserved residues, colon indicates residues with strongly similar properties and period indicates residues with weakly similar properties. The locations of AG1, AG4 and AG5 are indicated by black lines with critical residues of all epitope-mapped mAbs highlighted in red bolded letters. The positions of six IP-MS peptides (confidence level > 95%) identified in hANKRD1-expressing *E. coli* lysate using AG5/mAb 4F2 for antibody pull-down are shown (underlined in green). Two of the identified peptides overlap with their longer counterparts.

.

Supplementary Table 2. Checkerboard screening of antibody pairs against recombinant hANKRD1 (OriGene Technologies). The ratio of the absorbance (450nm) values between the positive and negative (identical to the positive test except that analyte is not added) control wells are shown. Values close to 1 indicate no difference in signal response between positive and negative control wells. Antibody pairs that give signal-to-noise ratios >10 are considered to be positive (highlighted in green boxes). In the case of positive antibody pairs, the second number (in brackets) represents the raw absorbance value of the positive well.

Capture \ Detector	AG1-30D12	AG1-8A3	AG4-13E10	AG4-4E1	AG4-10B12	AG4-5C1	AG4-1B1	AG5-4F2	AG5-27D7	AG5-2C9	AG5-6A4	AG5-11D11
**AG1-30D12**	54.9 (0.93)	2.98	1.03	1.00	1.01	1.09	1.15	30.4 (3.32)	1.19	7.09	3.31	2.17
**AG1-8A3**	5.07	1.14	0.90	1.02	1.06	1.00	0.95	13.3 (0.83)	1.06	7.52	2.25	1.83
**AG4-13E10**	1.29	0.99	1.00	0.99	0.93	0.91	0.96	1.13	0.99	1.09	1.06	1.00
**AG4-4E1**	1.13	1.03	1.00	0.99	0.94	0.95	1.04	1.12	1.04	1.01	1.06	1.00
**AG4-10B12**	4.47	1.13	0.97	0.98	0.95	1.00	0.96	2.78	1.03	1.09	1.13	1.04
**AG4-5C1**	44.7 (0.85)	3.22	1.00	0.98	0.97	1.03	1.04	17.6 (2.04)	1.11	3.08	1.62	1.44
**AG4-1B1**	41.8 (0.75)	3.12	0.99	0.98	0.99	0.96	1.00	16.7 (1.81)	1.06	2.98	1.44	1.33
**AG5-4F2**	62.9 (1.32)	3.02	0.99	1.00	1.04	1.06	1.03	11.84 (1.03)	1.05	1.49	1.25	1.13
**AG5-27D7**	4.81	1.46	0.99	1.00	1.01	1.09	1.00	2.59	1.04	1.07	1.18	1.04
**AG5-2C9**	40.1 (0.80)	2.27	1.01	0.99	1.03	1.14	1.10	12.3 (1.18)	0.99	1.60	1.36	1.24
**AG5-6A4**	50.4 (0.71)	3.38	1.00	1.01	1.06	1.17	1.08	11.5 (1.31)	1.05	2.07	1.67	1.31
**AG5-11D11**	43.3 (0.65)	3.16	1.00	1.00	1.01	1.04	1.04	11.8 (1.29)	1.06	2.04	1.67	1.36

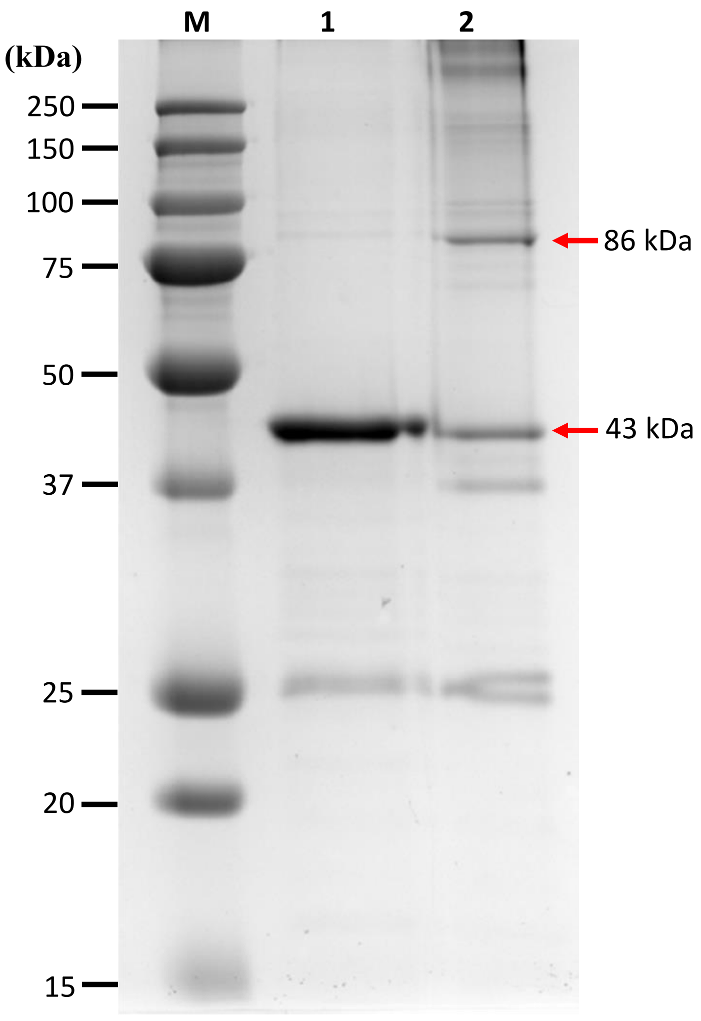

Supplementary Figure 6. SDS-PAGE analysis of recombinant hANKRD1 under reducing and non-reducing conditions. Recombinant hANKRD1 (OriGene Technologies) protein was prepared in reduced and non-reduced forms. The reduced sample was prepared by addition of SDS-protein dissociation sample buffer containing 5 % β-mercaptoethanol to the protein sample. The mixture was vortexed and then boiled at 95ºC for 10 minutes. For the preparation of the non-reduced protein sample, sample buffer without β-mercaptoethanol was added to the protein and mixed by vortexing for 2 minutes. The samples were then analyzed by 16% SDS-PAGE. Lane M, Precision Plus molecular weight marker (Bio-Rad); Lane 1, hANKRD1 (reducing conditions); Lane 2, hANKRD1 (non-reducing conditions). Monomeric hANKRD1 migrates to the 43-kDa position which is in agreement with its electrophoretic behavior as indicated in the manufacturer's product data sheet. Correspondingly, dimeric hANKRD1 was observed at the 86 kDa position under non-reducing conditions.

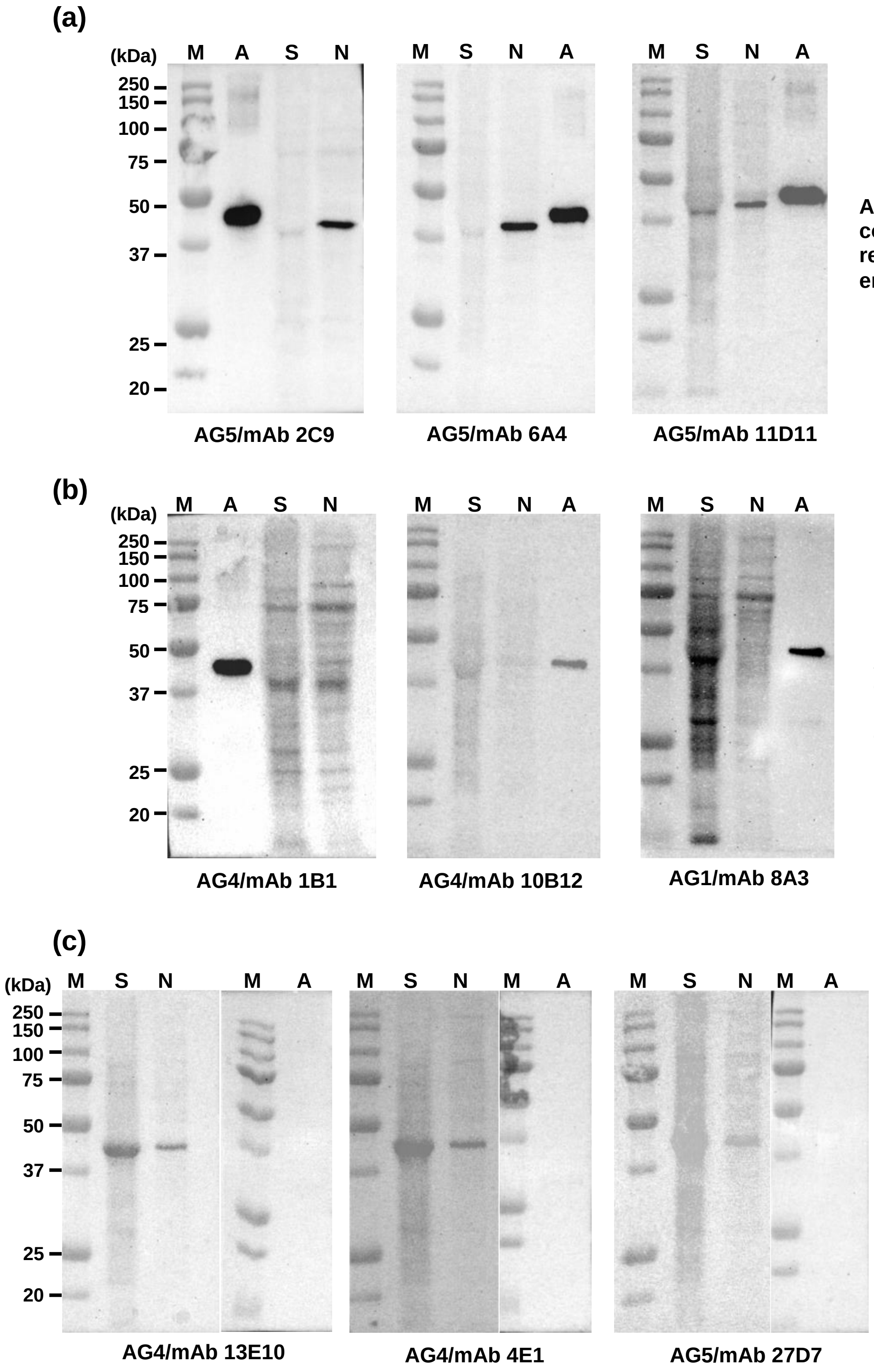

Antibodies that detect bands consistent with full-length recombinant hANKRD1 and endogenous rANKRD1.

Antibodies that detect bands consistent with full-length recombinant hANKRD1 but not endogenous rANKRD1.

Antibodies that do not detect bands consistent with full-length recombinant hANKRD1 or endogenous rANKRD1.

The positions of the detected bands (~ 43/44 kDa) are offset from the molecular weight positions of rANKRD1 determined in adult cardiac tissue (39.0±0.7 kDa) and neonatal cardiomyocytes (41.0±0.3 kDa).

Supplementary Figure 7. Antibody validation by Western blot analysis. Antibodies are grouped accordingly to their ability to detect bands consistent with full-length recombinant hANKRD1 (OriGene Technologies, A) and/or endogenous rANKRD1 in cell lysates obtained from adult rat heart tissue (Sham, S) and neonatal rat cardiomyocytes (N).

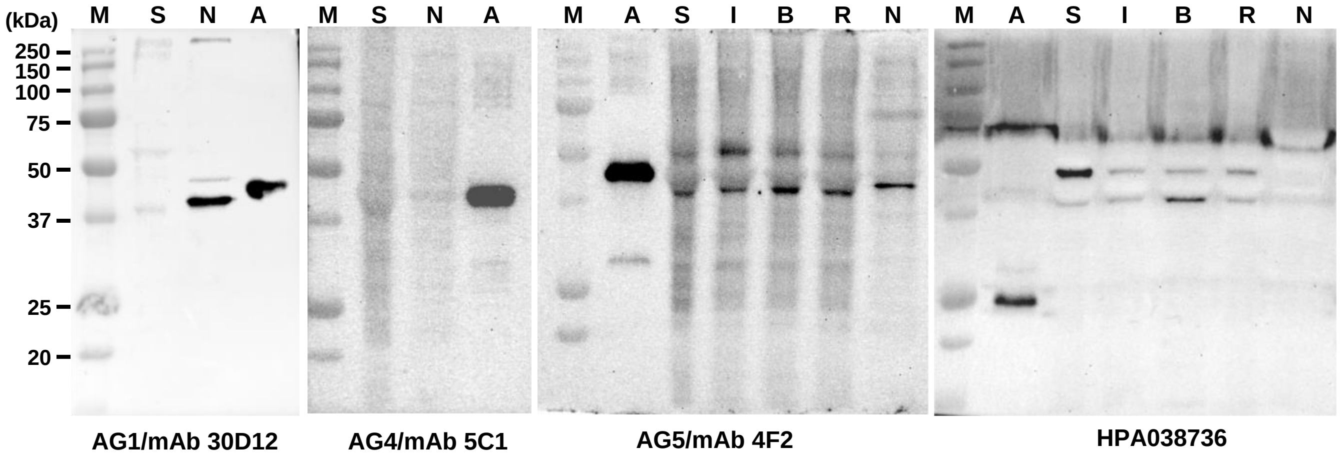

Supplementary Figure 8. Antibody validation by Western blot analysis. Immunoreactivity of mAbs (indicated below each panel) against recombinant hANKRD1 (A; 10 ng per well) and total protein (50 µg per well) isolated from whole cell lysates of adult rat heart tissue (Sham, S), myocardial infarcted Day 2 tissue (infarcted region, I; border region, B; remote region, R) and neonatal rat cardiomyocytes (N). The HPA038736 polyclonal antibody did not produce immunodetected bands of the expected 43 and 41 kDa sizes for recombinant hANKRD1 and neonatal rANKRD1, respectively. M, All-Blue Precision Protein Standards (BioRad).

a

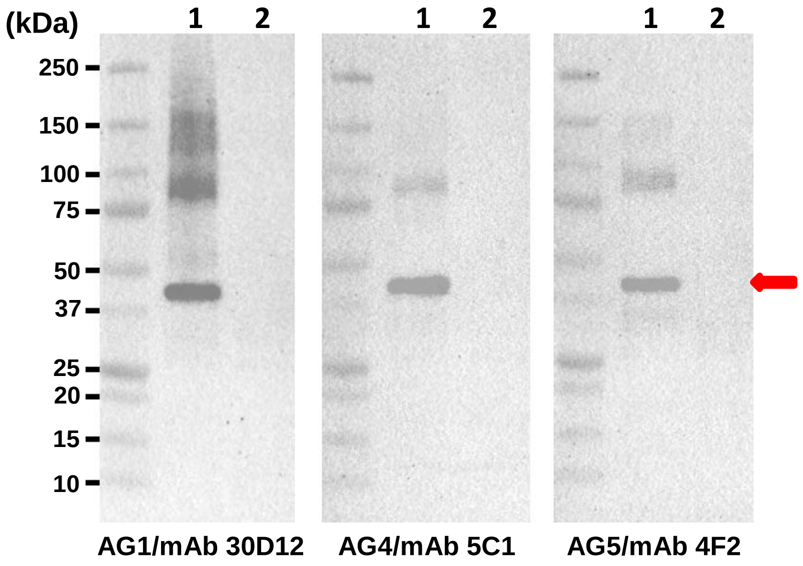

b

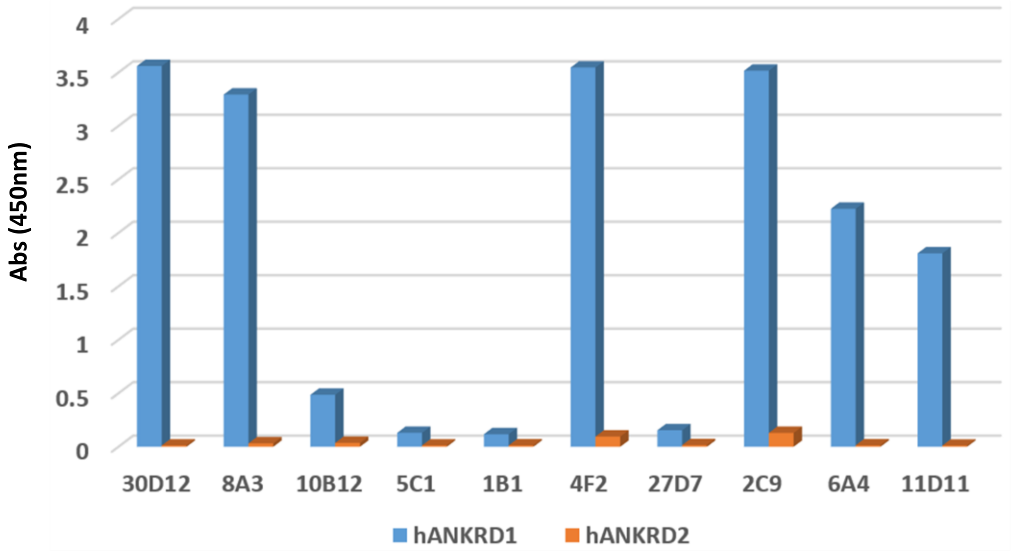

Supplementary Figure 9. Anti-hANKRD1 mAbs do not cross-react with hANKRD2. (a) Confirmatory Western blot showing the specificity of AG1/mAb 30D12, AG4/mAb 5C1 and AG5/mAb 4F2 against hANKRD1 (Lane 1) and not hANKRD2 (Lane 2). Recombinant hANKRD1 and hANKRD2 (TP760289 and TP310468 respectively; OriGene Technologies, Maryland, USA; 50ng each) were separated by SDS-PAGE and electroblotted onto an Immuno-Blot® PVDF membrane (BioRad Laboratories). Detection was performed using the purified mAbs (0.5 µg/ml) and a goat anti-mouse secondary antibody conjugated with horse radish peroxidase (diluted 1:2000; Agilent Technologies, USA). Protein bands were visualized using a chemiluminescence substrate (Thermo Fisher Scientific, USA). **(b)** Specificity testing of protein-reactive antibody panel against hANKRD1 and hANKRD2 by antigen-down assay. All mAbs gave absorbance values for hANKRD1 that were 10 – 285 times above the corresponding absorbance value for hANKRD2.

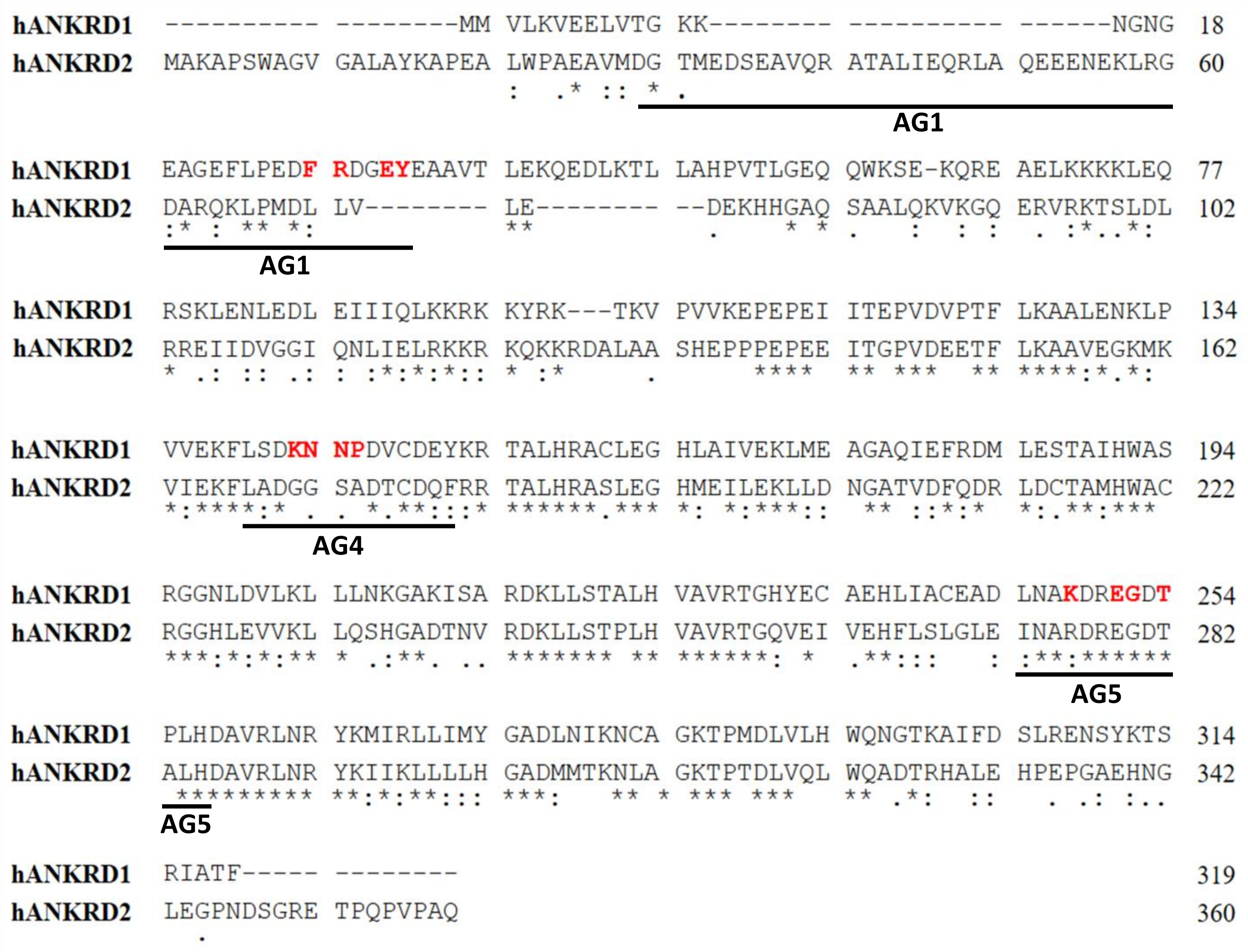

Supplementary Figure 10. Alignment of human ANKRD1 (GenBank Accession No. EAW50116.1) and the canonical cardiac isoform of ANKRD2 (Genebank Accession AAI07760.1) amino acid sequence. Sequence alignment was performed using Clustal Omega[5] available from the EMBL-EBI server[6] at https://www.ebi.ac.uk. An asterisk indicates fully conserved residues, blank indicates non-conserved residues, colon indicates residues with strongly similar properties and period indicates residues with weakly similar properties. The locations of AG1, AG4 and AG5 are indicated by black lines with critical residues of AG1/mAb 30D12, AG4/mAb 5C1 and AG5/mAb 4F2 highlighted in red bolded letters.

SUPPLEMENTARY REFERENCES

1. Liew, O.W., Ching Chong, J.P., Yandle, T.G. & Brennan, S.O. Preparation of recombinant thioredoxin fused N-terminal proCNP: Analysis of enterokinase cleavage products reveals new enterokinase cleavage sites. *Protein Expr. Purif.* **41,** 332–340 (2005).

2. Liew, O.W. et al. An SRLLR motif downstream of the scissile bond enhances enterokinase cleavage efficiency. *Biochimie* **89,** 21–29 (2007).

3. Liew, O.W. et al. A His6-SUMO-eXact tag for producing prepro-Urocortin 2 in *Escherichia coli* for raising monoclonal antibodies. *J. Immunol. Methods* **403,** 37-51 (2014).

4. Lau, C.Y., Ahmad Zahidi, A.A., Liew, O.W. & Ng, T.W. A direct heating model to overcome the edge effect in microplates. *J. Pharm. Biomed. Anal.* **102,** 199-202 (2015).

5. Sievers, F. et al. Fast, scalable generation of high-quality protein multiple sequence alignments using Clustal Omega. *Mol. Syst. Biol.* **7,** 539 (2011).

6. Chojnacki, S., Cowley, A., Lee, J., Foix, A. & Lopez, R. Programmatic access to bioinformatics tools from EMBL-EBI update: 2017. *Nucleic Acids Res.* **45(W1),** W550-W553 (2017).
