# Supplementary material for: Epitope-directed monoclonal antibody production using a mixed antigen cocktail facilitates antibody characterization and validation
Source: Commun Biol. 2021 Apr 6;4:441. doi: 10.1038/s42003-021-01965-x (PMC8024308; doi:10.1038/s42003-021-01965-x)
Supplement: Supplementary file 3 — Description of Supplementary Files [file 42003_2021_1965_MOESM3_ESM.pdf]

Description of Additional Supplementary Files

File name: Supplementary Data 1

Description: Excel file containing source data for Figures 3b, d and 4a-d.

File name: Supplementary Data 2

Description: Excel file containing source data from mass spectrometry analysis of mAb 4F2 pull-down material from lysate of E.coli expressing hANKRD1.
