# Supplementary material for: Epitope-directed monoclonal antibody production using a mixed antigen cocktail facilitates antibody characterization and validation
Source: Commun Biol. 2021 Apr 6;4:441. doi: 10.1038/s42003-021-01965-x (PMC8024308; doi:10.1038/s42003-021-01965-x)
Supplement: Supplementary file 6 — Reporting Summary [file 42003_2021_1965_MOESM6_ESM.pdf]

nature research

Corresponding author(s): Oi Wah Liew

Last updated by author(s): Feb 23, 2021

Reporting Summary

Nature Research wishes to improve the reproducibility of the work that we publish. This form provides structure for consistency and transparency in reporting. For further information on Nature Research policies, see our Editorial Policies and the Editorial Policy Checklist.

Statistics

For all statistical analyses, confirm that the following items are present in the figure legend, table legend, main text, or Methods section.

n/a	Confirmed	
☐	☒	The exact sample size (*n*) for each experimental group/condition, given as a discrete number and unit of measurement
☐	☒	A statement on whether measurements were taken from distinct samples or whether the same sample was measured repeatedly
☐	☒	The statistical test(s) used AND whether they are one- or two-sided<br>*Only common tests should be described solely by name; describe more complex techniques in the Methods section.*
☒	☐	A description of all covariates tested
☒	☐	A description of any assumptions or corrections, such as tests of normality and adjustment for multiple comparisons
☒	☐	A full description of the statistical parameters including central tendency (e.g. means) or other basic estimates (e.g. regression coefficient) AND variation (e.g. standard deviation) or associated estimates of uncertainty (e.g. confidence intervals)
☒	☐	For null hypothesis testing, the test statistic (e.g. F, t, r) with confidence intervals, effect sizes, degrees of freedom and P value noted<br>*Give P values as exact values whenever suitable.*
☒	☐	For Bayesian analysis, information on the choice of priors and Markov chain Monte Carlo settings
☒	☐	For hierarchical and complex designs, identification of the appropriate level for tests and full reporting of outcomes
☒	☐	Estimates of effect sizes (e.g. Cohen's d, Pearson's r), indicating how they were calculated

Our web collection on statistics for biologists contains articles on many of the points above.

Software and code

Policy information about availability of computer code

Data collection	Enspire microplate reader software was used to collect and analyse immunoassay data.
Data analysis	GraphPad QuickCalcs: t test calculator was used for group comparisons of experimental myocardial infarction (MI) model rats. Acquired mass spectrometry spectra were searched using the ProteinPilot 5.0 software (SCIEX)

For manuscripts utilizing custom algorithms or software that are central to the research but not yet described in published literature, software must be made available to editors and reviewers. We strongly encourage code deposition in a community repository (e.g. GitHub). See the Nature Research guidelines for submitting code & software for further information.

Data

Policy information about availability of data

All manuscripts must include a data availability statement. This statement should provide the following information, where applicable:

- Accession codes, unique identifiers, or web links for publicly available datasets
- A list of figures that have associated raw data
- A description of any restrictions on data availability

Most data generated or analysed in this study are included in the main text of this manuscript (and its supplementary information files). Raw data for microplate performance validation and ELISAs (Figure 3b, 3d, 4a-d) and mass spectrometry raw data are available in Supplemental data 1 and 2.

Field-specific reporting

Please select the one below that is the best fit for your research. If you are not sure, read the appropriate sections before making your selection.

☒ Life sciences ☐ Behavioural & social sciences ☐ Ecological, evolutionary & environmental sciences

For a reference copy of the document with all sections, see nature.com/documents/nr-reporting-summary-flat.pdf

Life sciences study design

All studies must disclose on these points even when the disclosure is negative.

Sample size	No sample size calculation was performed. The number of plates used to determine microplate performance was based on previous reported methodology (Lilyanna et al 2018, Journal of Applied Laboratory Medicine, Vol. 2(5), 687-699). The number of rats used in the experimental MI model was based on tissue and plasma samples that are available from our previous published study (Zhou et al. 2019, Molecular Therapy: Nucleic Acids, Vol 17, 185-197).
Data exclusions	No data was excluded.
Replication	Microplate well variability was evaluated on all 96 wells for each plate. This was performed at three different antigen coating concentrations and performance testing was done on 7 microplates per concentration. With limited plasma availability from each animal, measurement of rat plasma ANKRD1 was performed in duplicates in one assay. Intra-assay CV of all plasma ANKRD1 reported are less than 12%.
Randomization	Microplates were randomly picked from one batch lot for each plate type. Rat body weights were measured and cardiac function (ejection fraction) were assessed by echocardiography for all the animals at baseline. Surgery was scheduled for equal number of sham and LAD (left anterior descending coronary artery) ligation operations per day performed in random order.
Blinding	Blinding was not possible for the microplate performance evaluation because their physical characteristics are visually distinct. Blinding for all other experiments is not necessary as the focus of the study is on antibody utility/characterization/validation and not biological effects of treatment groups.

Reporting for specific materials, systems and methods

We require information from authors about some types of materials, experimental systems and methods used in many studies. Here, indicate whether each material, system or method listed is relevant to your study. If you are not sure if a list item applies to your research, read the appropriate section before selecting a response.

Materials & experimental systems

n/a	Involved in the study
☐	☒ Antibodies
☐	☒ Eukaryotic cell lines
☒	☐ Palaeontology and archaeology
☐	☒ Animals and other organisms
☒	☐ Human research participants
☒	☐ Clinical data
☒	☐ Dual use research of concern

Methods

n/a	Involved in the study
☒	☐ ChIP-seq
☒	☐ Flow cytometry
☒	☐ MRI-based neuroimaging

Antibodies

Antibodies used	The monoclonal antibodies used in this study were all developed in-house within the corresponding author's laboratory. The polyclonal antibody against full-length human ANKRD1 is from Atlas Antibodies (HPA038736; lot number A106040)
Validation	Validation of the monoclonal antibodies developed in this study is fully described in the main text and supplemental materials of this manuscript.

Eukaryotic cell lines

Policy information about cell lines

Cell line source(s)	The H9c2 cell line was obtained from ATCC (ATCC no. CRL-1466, lot number 60279340).
Authentication	Species authentication by ATCC as per Certificate of Analysis; our laboratory did not conduct any verification procedures ourselves.
Mycoplasma contamination	Mycoplasma contamination-free as confirmed in ATCC Certificate of Analysis obtained upon receipt of cell line. ATCC

	mycoplasma testing was based on Hoechst DNA stain (indirect) and agar culture (direct). Our laboratory did not conduct any mycoplasma testing ourselves.
Commonly misidentified lines (See ICLAC register)	No misidentified cell line in this study.

Animals and other organisms

Policy information about studies involving animals; ARRIVE guidelines recommended for reporting animal research

Laboratory animals	Mice used for monoclonal antibody production are Balb C female 8-week old animals. Male Sprague Dawley rats (8 weeks old) were used for the experimental MI model.
Wild animals	No wild animals were used in this study.
Field-collected samples	The study did not involve field collected samples.
Ethics oversight	Mouse immunization were performed in compliance with A*STAR Institutional Animal Care and Use Committee (IACUC) regulations. Rat MI protocol was approved by the Institutional Animal Care and Use Committee of the National University of Singapore and complied with the Guide for the Care and Use of Laboratory Animals published by the National Institutes of Health (NIH Publication No. 85–23, Revised 1996).

Note that full information on the approval of the study protocol must also be provided in the manuscript.
